# Machine Learning to Predict Brain Amyloid Pathology in Pre-dementia Alzheimer’s Disease Using QEEG Features and Genetic Algorithm Heuristic

**DOI:** 10.3389/fncom.2021.755499

**Published:** 2021-11-11

**Authors:** Nam Heon Kim, Dong Won Yang, Seong Hye Choi, Seung Wan Kang

**Affiliations:** ^1^iMediSync Inc., Seoul, South Korea; ^2^Department of Neurology, St. Mary’s Hospital, Seoul, South Korea; ^3^Department of Neurology, Inha University School of Medicine, Incheon, South Korea; ^4^National Standard Reference Data Center for Korean EEG, Seoul National University College of Nursing, Seoul, South Korea

**Keywords:** EEG, Alzheimer’s disease (AD), beta-amyloid, machine learning, diagnosis, genetic algorithm, pre-dementia Alzheimer’s disease

## Abstract

The use of positron emission tomography (PET) as the initial or sole biomarker of β-amyloid (Aβ) brain pathology may inhibit Alzheimer’s disease (AD) drug development and clinical use due to cost, access, and tolerability. We developed a qEEG-ML algorithm to predict Aβ pathology among subjective cognitive decline (SCD) and mild cognitive impairment (MCI) patients, and validated it using Aβ PET. We compared QEEG data between patients with MCI and those with SCD with and without PET-confirmed beta-amyloid plaque. We compared resting-state eyes-closed electroencephalograms (EEG) patterns between the amyloid positive and negative groups using relative power measures from 19 channels (Fp1, Fp2, F7, F3, Fz, F4, F8, T3, C3, Cz, C4, T4, T5, P3, Pz, P4, T6, O1, O2), divided into eight frequency bands, delta (1–4 Hz), theta (4–8 Hz), alpha 1 (8–10 Hz), alpha 2 (10–12 Hz), beta 1 (12–15 Hz), beta 2 (15–20 Hz), beta 3 (20–30 Hz), and gamma (30–45 Hz) calculated by FFT and denoised by iSyncBrain^®^. The resulting 152 features were analyzed using a genetic algorithm strategy to identify optimal feature combinations and maximize classification accuracy. Guided by gene modeling methods, we treated each channel and frequency band of EEG power as a gene and modeled it with every possible combination within a given dimension. We then collected the models that showed the best performance and identified the genes that appeared most frequently in the superior models. By repeating this process, we converged on a model that approximates the optimum. We found that the average performance increased as this iterative development of the genetic algorithm progressed. We ultimately achieved 85.7% sensitivity, 89.3% specificity, and 88.6% accuracy in SCD amyloid positive/negative classification, and 83.3% sensitivity, 85.7% specificity, and 84.6% accuracy in MCI amyloid positive/negative classification.

## Introduction

Dementia is a fatal disorder characterized by progressive decline in two or more cognitive abilities including memory, language, executive and visuospatial functions, personality, and behavior (Braak and Braak, 1995). Alzheimer’s disease (AD) is the most common cause (nearly 70%) of dementia worldwide. AD is accompanied by the accumulation of β−amyloid plaques and neurofibrillary tangles of hyperphosphorylated tau protein, causing progressive neurodegeneration in specific brain regions (Hyman et al., 2012; Kang et al., 2015).

Alzheimer’s disease is difficult to diagnose in its early stages because the cognitive decline can be subtle. Beta-amyloid is a well-characterized diagnostic index of AD and its accumulation can be used to predict progression from mild cognitive impairment (MCI) to dementia (Schipke et al., 2012). In many countries, drugs for AD can only be prescribed after beta-amyloid has been detected (Seubert et al., 2000; van Rossum et al., 2012). Although cerebrospinal fluid and positron emission tomography (PET) biomarkers, combined with relatively new clinical criteria, can help diagnose AD, they are both invasive and costly (Dubois et al., 2014).

Electroencephalograms (EEG) detect electrical activity in the brain using multiple scalp electrodes. Previous studies have identified several EEG patterns characteristic of specific brain diseases (Zhao and He, 2015; Siuly et al., 2016). Several studies have identified specific EEG patterns that may differentiate AD (Jeong, 2004) or MCI patients (Jelic et al., 1998, 2000; Huang et al., 2000; Grunwald et al., 2001) from normal subjects, although their statistical power was weak due to small sample sizes. Lehmann et al. (2007) had some success in classifying AD and normal subjects using an artificial neural network (ANN) with EEG features (Lehmann et al., 2007). Buscema et al. (2007) achieved approximately 92% accuracy in AD and MCI classification using EEG markers as inputs to ANN (Buscema et al., 2007), while Dauwels et al. (2009) reached 83 and 88% accuracy in classifying pre-dementia and mild AD, respectively. Stomrud et al. (2010) found that relative theta power was correlated with CSF tau/beta-amyloid ratio (Stomrud et al., 2010). Mander et al. (2015) found that non-REM sleep slow wave activity was correlated with beta-amyloid burden (Mander et al., 2015). More recently, Babiloni et al. (2017) achieved 75.5% accuracy in classifying AD patients and normal subjects based on features such as EEG power ratios, low-resolution brain electromagnetic tomography (LORETA), and coherences. Meanwhile, Farina et al. (2020) tried to classify AD, MCI, and healthy subjects using EEG combined MMSE score but no benefit was found relative to MMSE score alone (Farina et al., 2020). Smailovic et al. (2018) investigated correlations of quantitative EEG and synchronization with CSF biomarkers. And Michels et al. (2021) investigated EEG-fMRI and amyloid burden.

Recently, there have been various challenges to find AD biomarkers using less invasive biomarkers. Shen et al. (2020) used plasma biomarker to identify amyloid, tau, or neurodegeneration. Stockmann et al. (2020) designed a panel of structure-based Aβ plasma biomarkers as a prognostic tool for future progression from SCD to MCI or AD. Buegler et al. (2020) built digital biomarker-based prognostic models which predicted the risk to progress to dementia within 3 years. Those digital biomarker-based models are reviewed by Cavedoni et al. (2020).

Although previous studies have noted significant differences in EEG patterns among AD, MCI, and normal subjects, suggesting the capacity for accurate classification and the potential of EEG biomarker refinement for neurodegenerative diseases, none have succeeded in the development of a reliable EEG biomarker for beta-amyloid plaque accumulation. The present study describes our application of machine learning methods to provide high classification accuracy in discriminating the presence or absence of these plaques in pre-dementia patients.

A genetic algorithm is a search heuristic method inspired by the theory of natural evolution that has wide applications across science, industry, and beyond. It is especially suited to solving complex problems. The search begins with the best answer for a problem being expressed in a set form of data structure, which is gradually transformed to produce increasingly useful answers. It is based on the logic that competition between models containing various genes will gradually increase the proportion of genes included in the best models, ultimately leading to a model that contains only the most suitable genes for maximizing survival. Genetic algorithms have been shown to produce optimal combinations of features at relatively low computation costs (Babatunde et al., 2014).

## Research Method

### Materials

The present study employed a multicenter cohort design using EEG data from patients diagnosed with subjective cognitive decline (SCD) or MCI, each of which was also assessed using amyloid PET scans. Table 1 presents the distribution of subjects by institution and diagnosis. We randomly selected 20% of subjects (*N* = 28; 7 SCD amyloid positive, 28 SCD amyloid negative, 6 MCI amyloid positive, and 7 MCI amyloid negative) to exclude from the model training so they could be used for later verification. Since the ratio of positive to negative data was markedly skewed, being approximately 1:2.5, the positive data were doubled to balance the data set. As an augmentation method, each of the positive data were divided into the first half and the second half and treated as two separate set. We confirmed the split-half comparisons of each channel of each EEG data set was over 0.90.

**TABLE 1:** Number of subjects by institution, diagnosis, age, and gender.

	**SCD (+)**	**SCD (−)**	**MCI (+)**	**MCI (−)**	**Total**
Institution A	16	69	9	14	108
Institution B	18	77	20	20	135
Age (mean ± sd)	72.0 ± 5.9	71.3 ± 6.9	74.5 ± 6.1	71.5 ± 6.8	71.8 ± 6.7
Gender (M/F)	18/16	94/52	18/11	15/19	145/98
Total	34	146	29	34	243

*SCD (+), SCD (subjective cognitive decline) positive, amyloid PET positive; SCD (−), SCD positive, amyloid PET negative; MCI (+), MCI (mild cognitive impairment) positive, amyloid PET positive; MCI (−), MCI negative, amyloid PET negative.*

#### Diagnostic Criteria for Subjective Cognitive Decline and Mild Cognitive Impairment

The SCD inclusion criteria were (1) persistent subjective complaints of cognitive decline, (2) ≥ 60 years of age, (3) at least 6 years of primary school, (4) a memory test score below the normative mean by 0–1.5 standard deviations (SD), and deficits of ≤1.5 SD on the other cognitive tests used, and (5) informed consent of the participant. Individuals showing a deficit of >1.5 SD on any cognitive test were excluded because of the possibility of MCI. This is commonly used standard in Korean Alzheimer’s disease society (Ho and Yang, 2020).

We defined MCI using Petersen’s criteria, which presumes MCI in patients with objective memory impairment for their age, but normal performance in activities of daily living (ADL) (Petersen, 2004).

#### Diagnostic Criteria for Amyloid Positron Emission Tomography Result

Amyloid PET scans were performed to detect amyloid beta (Aβ) plaques in the brain. Cortical Aβ was quantified using the standardized uptake value ratio (SUVR) normalized to the cerebellar gray matter. ^18^F-florbetaben PET images were acquired and processed by the precedent method. Individual 3D T1-weighted magnetic resonance (MR) images were preprocessed and co-registered into the corresponding PET images. The MR images normalized to a standardized stereotaxic space were divided into three probabilistic tissue maps composed of gray matter, white matter, and cerebrospinal fluid. A volume-based template of the 90 regions-of-interest was aligned to the individual MR image. The SUVR was calculated using whole voxels of ^18^F-florbetaben PET images referenced to the cerebellum. The global SUVR was estimated by averaging 90 regional uptake values.

### Electroencephalograms Recordings

All subjects were instructed to relax with their eyes closed and to refrain from movement and talking. EEG data were recorded (bandpass: 0.1–45.5 Hz; Natus Nicolet EEG v32, Nihon Kohden JE921A, and Grass AS40) in the resting-state, eyes-closed condition from 19 scalp electrodes positioned over the whole head according to the International 10–20 System (Fp1, Fp2, F7, F3, Fz, F4, F8, T3, C3, Cz, C4, T4, T5, P3, Pz, P4, T6, O1, O2). A linked-ear reference electrode was noted if present, but not deemed mandatory for the present study, to respect standard internal protocols of the several clinical recording units. In each case, a ground electrode was located between the Afz and Fz electrodes. Electrode impedance was kept below 10 kOhm. All recorded artifact-free EEG data were re-referenced offline to a common average to harmonize the EEG data collected using different reference electrodes. All data were digitalized in continuous recording mode (approximately 3 min of EEG; sampling rate: 200 or 250 Hz, to avoid aliasing).

### Feature Generation

In each case, the relative power was used for the features. First, the power spectral density of the EEG rhythms was computed using an FFT analysis with 0.25 Hz of frequency resolution using the iSyncBrain^®^ AI-driven auto-analysis system. Then, the signal was decomposed into the following frequency bands: delta (1–4 Hz), theta (4–8 Hz), alpha 1 (8–10 Hz), alpha 2 (10–12 Hz), beta 1 (12–15 Hz), beta 2 (15–20 Hz), beta 3 (20–30 Hz), and gamma (30–45 Hz). For each channel and frequency band, the average power during the recoding was calculated and treated as a feature. This yielded 152 features in total (19 channels × 8 frequency bands).

### Feature Selections

To successfully predict amyloid positive or negative cases, among the many features associated with amyloid-positive EEG data, we selected those in which the change was most noticeable in the channel, frequency. In many previous machine learning studies, features were selected using various statistical analysis methods to identify those showing significant differences between the target group and the control group. Firstly, we conducted paired *t*-test to find statistically different features between amyloid positive and negative group. Additionally, the present study adopted a kind of exhaustive approach combined with a heuristic genetic search algorithm.

We attempted to exhaustively classify based on every combination of features. Thus, if we used one feature for classification, there were 152 cases. If we used a two-dimensional combination of features exhaustively, there were 11,476 cases (152 × 151/2), while a three-dimensional combination of features would yield 573,800 cases (152 × 151 × 150/3 × 2). We calculated the accuracy, sensitivity, and specificity for every case. However, there would be 21,374,050 (152 × 151 × 150 × 149/4 × 3 × 2) cases in a four-dimensional combination, and 632,671,880 (152 × 151 × 150 × 149 × 148/5 × 4 × 3 × 2) cases in a five-dimensional combination. To reduce the computation time this would demand, we used a genetic algorithm search heuristic to reduce the number of features.

**FIGURE 1:**
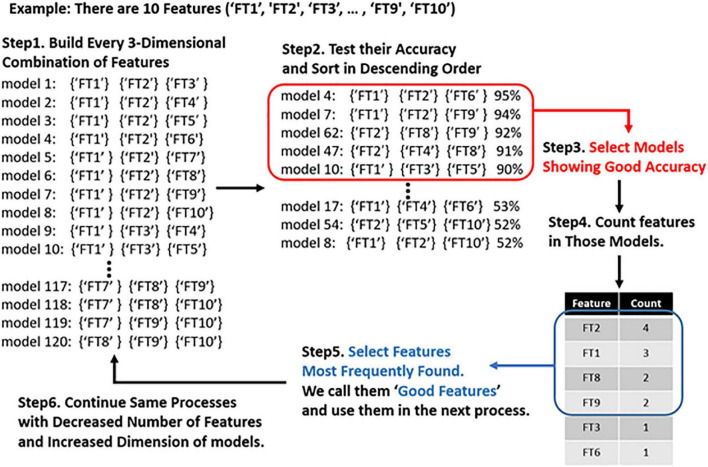
Schematic plot of feature selection with genetic algorithm. First, we make models using all three-dimensional combinations of features. Then we compute the accuracy of each model and identify the genes (EEG features) frequently present in the highest-performing model. We only use the best-performing genes to create a generation of models that are one dimension higher.

From classifications using a two-dimensional combination and three-dimensional combination of features, we collected the best combinations that showed the highest accuracy, and then identified the features that were frequently included in those “good models.” We assumed that the more frequently a feature is found in a “good model,” the more important the feature is to the desired output. Thus, we used the “good features” to build a four-dimensional combination with them, rather than using all 152 features. We then repeated this process using five-dimensional models. Figure 1 represents the schematic plot of feature selection with genetic algorithm heuristic.

### Machine Learning Models

The significant feature sets obtained by the feature selection procedure were then entered into representative machine learning models, SVM, and the accuracy was calculated by fivefold cross-validation. The feature set that showed more than 75% of accuracy in the above verification was used in the production of this model as an important feature set.

### Evaluation of Models

We input the test data (13 positive and 35 negative cases) which had been set aside for further validation. The final performance of each model was determined by the result of the test validation. The models with the highest accuracy were selected as the final model candidates.

## Results

### Feature Selection

**FIGURE 2:**
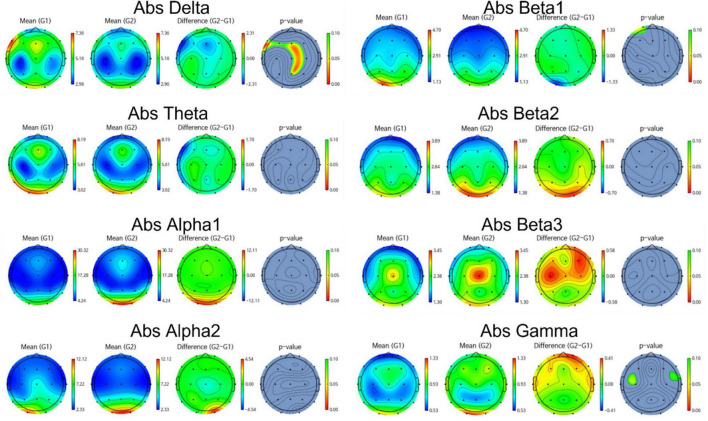
Topomap showing group difference in EEG absolute power between amyloid positive and negative.

**FIGURE 3:**
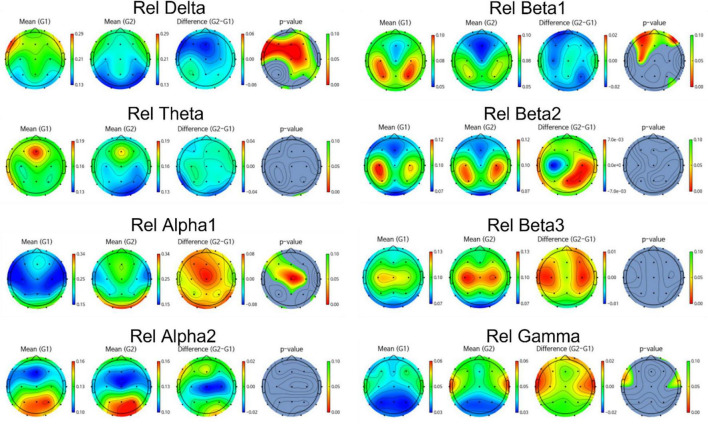
Topomap showing group difference in EEG relative power between amyloid positive and negative.

Group differences in EEG between amyloid positive and negative were calculated by *t*-test. Figures 2, 3 illustrate group differences calculated by t-test in EEG absolute power, and relative power respectively.

EEG power of amyloid positive group was stronger than amyloid negative group in following frequency bands and channels:

absolute power—delta F3, F7, Fz, Cz, C4,Pz, relative power—delta Fp2, F3, Fz, F4, F7, C3, Cz, C4, Pz, P4, T6, O1, and beta1 Fp1, Fp2, F3, Fz, F4, C3. On the other hand, EEG power of amyloid positive group was weaker in following regions: absolute power—gamma C3, T4, relative power—alpha Fp1, F3, Fz, C3, Cz, C4, Pz, T6, and gamma Fp2, F7, F8, C4, T3, T4.

Meanwhile, most highly evaluated features selected by genetic algorithm were as follows: Absolute power -delta F4, Fz, C4, Pz, alpha1 Fp1, Fp2, beta2, C3, T5, P3, beta3 C3, T5, gamma F7, F3, C3, T5 and relative power—delta C3, C4, F7, Cz, T6, P3, Pz, P4, O1 theta T4, P3, alpha1 F3, T4, T5, Fz, F4, F7, alpha2 F3, Fz, F4, O1, O2, beta1 F4, C3, C4, O1, O2, beta2 F3, F7 and gamma F8, C4, O2.

Every 5-dimentional models were built combining 5 of those features. We performance of each model and choose 20 models which showed high accuracy both in cross validation and test data.

**FIGURE 4:**
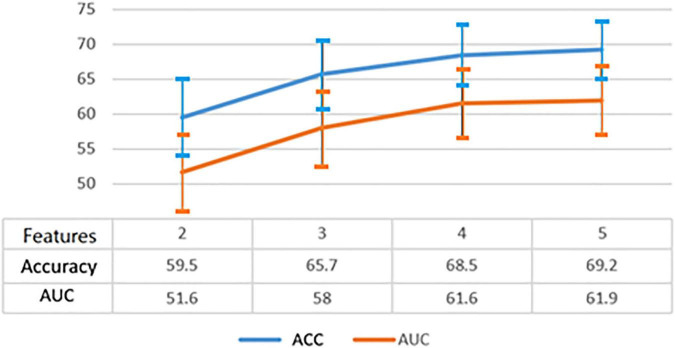
Model performances with different number of features.

Figure 4 shows the average performance of created models with different number of features. We confirmed that as the dimensions of features increased, we could decrease the number of features considered good and thereby improve the average performance of the models. The best models attained over 80% accuracy. We identified the features that were most frequently included in these models, and used them to create higher-dimension models.

### Multi-Model Ensembles and Weighting

The above-described process yielded multiple models with different features, each of which achieved over 80% accuracy. To make the final model more flexible and build a scoring system, we combined multiple models that were designed to classify amyloid positive or negative cases. For example, the final (SCD amyloid positive vs. SCD amyloid negative) classifier consists of 20 sub-models. If all 20 sub-models predicted the unknown data as positive, the score would be 1.0; if half the models predicted the data as positive, the score would be 0.5; and if none of the models predicted the data as positive, the score would be 0. Table 2 illustrates a schematic for composition of multiple sub-models and the scoring system.

**TABLE 2:** A schematic for composition of multiple sub-models and the scoring system.

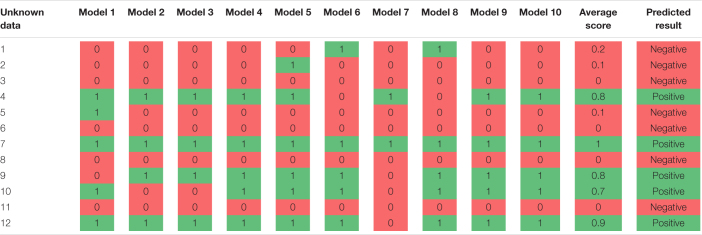

### Final Performance

**FIGURE 5:**
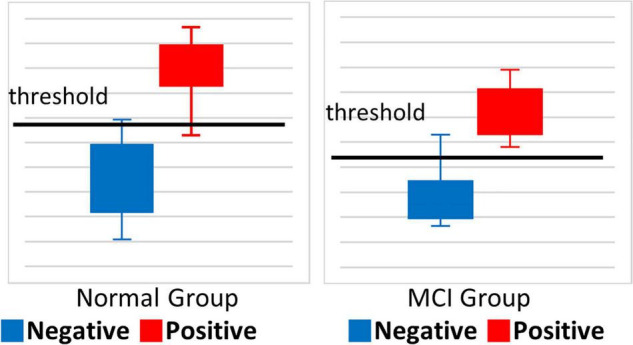
Box plot of amyloid classification score.

Figure 5 illustrates how the model scored amyloid negative samples and amyloid positive samples. The cutoff score for SCD was 0.58 and that for MCI was 0.45.

#### Classification

The best SCD model achieved 85.7% sensitivity, 89.3% specificity, and 88.6% accuracy. The best MCI model achieved 83.3% sensitivity, 85.7% specificity, and 84.6% accuracy. Balanced accuracy for the best SCD model was 87.5% and for the best MCI model was 84.5%. The confusion matrix of classification is shown in Table 3.

**TABLE 3:** Confusion matrix of classification.

**SCD**	**True positive**	**True negative**	**MCI**	**True positive**	**True negative**
Predicted positive	6	3	Predicted positive	5	1
Predicted negative	1	25	Predicted negative	1	6

## Conclusion

### Overview

We collected resting state eyes-closed EEG rhythms in pre-dementia subjects, each of whom also underwent amyloid PET analysis. We aimed to discriminate and classify an amyloid positive pre-dementia group and amyloid negative pre-dementia group from their EEG features alone. While several previous studies have investigated differences in the EEG power patterns between amyloid positive and negative patients, none have confirmed or quantified the plaque at a molecular level. Thus, we have only had correlational data relating amyloid plaque and EEG patterns (Smailovic et al., 2018).

An additional limitation has been the complexity of the feature sets used, which render analysis too challenging for traditional statistical approaches. To address this challenge, we performed feature selection using a genetic algorithm strategy to identify the best combinations of channels and frequency bands of EEG power to achieve the most accurate classification. While this is a relatively exhaustive method, and is more substantive than statistical analysis alone, there remain limitations. In our approach, as the dimension of features increases, we collect better features for the next dimension in order to reduce the computational cost. Therefore, it is not fully exhaustive, and there is the possibility of missing better combinations. Furthermore, there is also the possibility of overfitting.

Currently, one of the most commonly used tests for AD diagnosis is the amyloid PET scan, since amyloid plaque is a specific marker of AD. However, amyloid PET is costly and carries risks associated with radiation exposure, both of which limit its applications. There is general agreement that it is important to detect AD in its earliest stages, to both reduce healthcare costs and to halt or slow its progression. We chose QEEG to assist in early detection, since it is far less expensive, readily available, non-invasive, and safe.

The present findings suggest that accurate classification for beta-amyloid accumulation in the brain based on QEEG alone is possible, which implies that QEEG is a promising biomarker for beta-amyloid. Since QEEG is more accessible, cost-effective, and safer than amyloid PET, QEEG-based biomarkers may play an important role in the diagnosis and treatment of AD. Further, our findings suggest that genetic algorithms can be useful for feature selection in QEEG, greatly reducing the number of relevant feature combinations among brain signals.

### Future Work

To address the overfitting problems inherent in studies using limited numbers of subjects within a single institution, we are now pursuing more third-party data from diverse institutions in our validation efforts. It is noteworthy that several EEG studies have used a variety of EEG features in addition to band power, such as power ratios, sLORETA, and functional connectivity.

We also plan to more closely investigate the subset of misclassified subjects from the present study to identify specific patterns and methods they may have contributed to these errors. We will also continue to develop features such as band power ratio, sex- and age-matched normative z-score power, source cortical activity from standardized low-resolution brain electromagnetic tomography (sLORETA), and their correlations. One especially promising approach is to apply deep learning using graphically represented QEEG data to permit analysis via an ANN. Using these and other approaches, we expect to extend our capabilities toward the classification of beta-amyloid plaque with more than 80% accuracy, regardless of the patient’s diagnosis: MCI or SCD.

## Data Availability Statement

The original contributions presented in the study are included in the article/supplementary material, further inquiries can be directed to the corresponding author.

## Ethics Statement

The studies involving human participants were reviewed and approved by the Inha University Hospital Ethics Committee and Catholic University Hospital Ethics Committee. The patients/participants provided their written informed consent to participate in this study.

## Author Contributions

NK mainly did the data analysis, feature engineering, machine learning modeling, and manuscript writing. DY and SC mainly collected the data, determined the diagnosis, and provided the clinical advice. SK designed this project, provided the data, equipment, and lots of advice. All authors contributed to the article and approved the submitted version.

## Conflict of Interest

NK and SK report personal fees from iMediSync Inc. The remaining authors declare that the research was conducted in the absence of any commercial or financial relationships that could be construed as a potential conflict of interest.

## Publisher’s Note

All claims expressed in this article are solely those of the authors and do not necessarily represent those of their affiliated organizations, or those of the publisher, the editors and the reviewers. Any product that may be evaluated in this article, or claim that may be made by its manufacturer, is not guaranteed or endorsed by the publisher.
